# Cell Death and Deubiquitinases: Perspectives in Cancer

**DOI:** 10.1155/2014/435197

**Published:** 2014-07-09

**Authors:** Seemana Bhattacharya, Mrinal Kanti Ghosh

**Affiliations:** Signal Transduction in Cancer and Stem Cells Laboratory, Division of Cancer Biology and Inflammatory Disorder, Council of Scientific and Industrial Research-Indian Institute of Chemical Biology (CSIR-IICB), 4, Raja S.C. Mullick Road, Jadavpur, Kolkata 700 032, India

## Abstract

The process of cell death has important physiological implications. At the organism level it is mostly involved in maintenance of tissue homeostasis. At the cellular level, the strategies of cell death may be categorized as either suicide or sabotage. The mere fact that many of these processes are programmed and that these are often deregulated in pathological conditions is seed to thought. The various players that are involved in these pathways are highly regulated. One of the modes of regulation is via post-translational modifications such as ubiquitination and deubiquitination. In this review, we have first dealt with the different modes and pathways involved in cell death and then we have focused on the regulation of several proteins in these signaling cascades by the different deubiquitinating enzymes, in the perspective of cancer. The study of deubiquitinases is currently in a rather nascent stage with limited knowledge both *in vitro* and *in vivo*, but the emerging roles of the deubiquitinases in various processes and their specificity have implicated them as potential targets from the therapeutic point of view. This review throws light on another aspect of cancer therapeutics by targeting the deubiquitinating enzymes.

## 1. Introduction 

The balance between cell division and cell death is very important to coordinate normal cell turnover in both development and maintenance of tissue homeostasis in multicellular organisms [1]. The process of cell death has been selected evolutionarily over the years as an integral cellular mechanism [2] and any deregulation may lead to irregularities in embryogenesis, neurodegenerative disorders, and development of cancer [1]. Cell death may occur both normally or under certain pathological conditions. The existence of multiple death pathways is an in-built strategy to protect the organism against abnormalities arising in a single or multiple pathways, making the occurrence of diseases like cancer relatively rare, considering the large number of cell divisions and mutations incorporated during the lifetime of a multicellular organism [1].

With the advances in recent research over the last two decades new insights into the mechanisms and the factors involved in cell death have emerged, increasing its importance with respect to diseases. At the molecular level, cell death involves DNA damage, mitochondrial cytochrome c release, endoplasmic reticulum (ER) stress response, and so forth. The classification of the cellular death pathways is not discrete at certain instances and due to the presence of overlapping signaling pathways regulating these processes, there are a number of common factors involved, making the classification ambiguous. More than one death program may be activated at the same time and also there may be switching from one pathway to another depending on the context [3]. However, the basic mechanisms of cell death may be broadly classified as either suicide or sabotage [4]. In programmed cell death, the ultimate fate of the cell depends on the initiation signal and the degree of assault, and the outcome is known.

The programmed cell death processes involve well-coordinated factors and hence are subject to various modes of regulation. As in all cases the regulation may be at the gene, mRNA, or protein level. In this review, we focus on the post-translational modifications of proteins, specifically deubiquitination. We know that the process of ubiquitination is extremely dynamic, involving transient protein-protein interactions with high specificity and functions in a highly context-dependent manner. Ubiquitination may be monomeric, polymeric, or multimonomeric, involving a variety of ubiquitin (Ub) linkages such as Lys 6 (K6), K11, K27, K29, K33, K48, K63, and Met1, each having specific functions [19]. This is a reversible process, involving deubiquitinating enzymes (DUBs) [20]. There are about 100 genes encoding DUBs in the human genome and this large number strongly suggests that distinct enzymes have highly specialized functions, but this study is still in a very nascent stage [21]. The basic features of DUB activity are processing of Ub precursors, editing of Ub chains, reversal of Ub conjugation, and recycling of Ub [22]. The specificity and activity of the DUBs is ensured by protein-protein interactions, the multiprotein complexes with which DUBs are associated, subcellular localization, phosphorylation, and changes in their expression or even differential activity in the various phases of cell cycle [21, 23]. Deubiquitinases regulate a variety of cellular processes by reversing ubiquitination.

DUBs are classified into six families: ubiquitin carboxy-terminal hydrolases (UCHs), ubiquitin specific proteases (USPs), ovarian-tumor proteases (OTUs), JAMM/MPN domain-associated metallopeptidases (JAMMs), Machado-Joseph disease protein domain proteases (MJDs), and monocyte chemotactic protein-induced proteases (MCPIPs). All these enzymes are cysteine proteases except the JAMMs. The largest family is the USPs with more than 50 members, all containing conserved domains and catalytic sites [20]. DUBs are often deregulated in cancers showing either mutations or altered expression levels [24]. Moreover, cancer cells are more sensitive to defects in protein folding and stability and E3 ligases are already being targeted for therapeutic purposes (e.g., bortezomib). But the DUBs are comparatively lesser in number and much more specific with respect to their functions and, hence, likely to be better targets [23, 24]. Recent research is bringing up first-generation inhibitors with specificity against either a single or a related group of DUBs [25]. Hence, the growing interest and importance of targeting DUBs bring the study of DUBs to high priority. In this review, we have tried to outline briefly the various programmed cell death pathways and their players and have also emphasized on the various deubiquitinases that are associated with these factors, focusing mainly on the context of cancer.

## 2. Importance of Cell Death in Cancer

### 2.1. Modes of Cell Death

**Figure 1:**
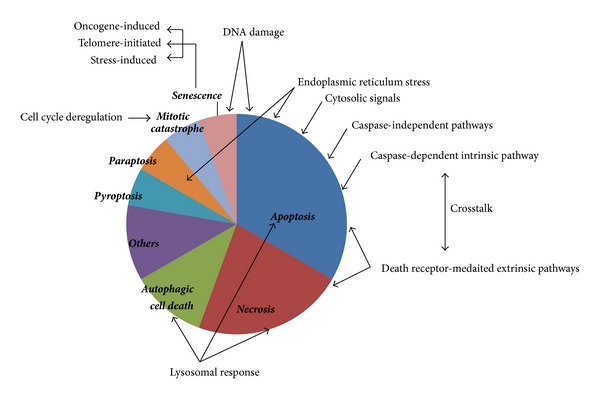
Relative occurrence of the various modes of programmed cell death. The figure illustrates the relative occurrence of different modes of programmed cell death (in bold and italics). The signaling mechanisms that are triggered as a cellular response leading to a specific mode of cell death are also represented in the figure (normal text and arrows pointing towards the process).

The three main programmed cell death mechanisms are apoptosis, necrosis and autophagic cell death [26]. Several other modes of cell death have been reported such as paraptosis and mitotic catastrophe (see Figure 1). The different modes of cell death are not discrete at all times and there are frequent crosstalks. The outcomes may be a shared response of different modes of cell death but each one has its own salient features as described below. A comparative analysis of the different modes is illustrated in Table 1.

*Apoptosis.* Apoptosis may occur during embryonic development, in mature tissues like thymus or under pathological conditions. The cardinal features of apoptosis include membrane blebbing, rounding up of cells, reduction of cell volume, chromatin condensation, and nuclear fragmentation. This follows either a caspase-dependent or caspase-independent pathway, which may or may not be associated with mitochondrial and/or immunological involvement, based on intrinsic or extrinsic cues.

*Necrosis.* Necrosis generally occurs as a response to physical cellular injury and is mostly associated with pathological conditions. Necrosis is characterized by gain in cell volume, swelling of organelles, rupture of plasma membrane, and elicitation of inflammatory tissue response. Necrosis was initially thought to be an uncontrolled (accidental) death process, but recent evidences of well-defined signaling pathways involved in necrosis are coming into focus. Thus, programmed necrosis also known as “necroptosis,” exists as a back-up system for the cell when apoptosis is inhibited [27].

*Autophagic Cell Death.* Autophagy is a prosurvival strategy for the cells in cases of stress like nutrient or growth factor deprivation or cytokine-induction. The mode of action is via sequestration of cytoplasmic material within autophagosomes for lysosomal degradation, with the absence of chromatin condensation, generally mediated by the autophagy genes (ATGs) [4, 28]. Hence, activation of autophagy under cellular stress has a cytoprotective outcome to maintain cellular homeostasis and inhibiting it may lead to cell death. This may again cause inhibition of developmental cell death indicating a role of autophagy in cell death. Therefore, the decision of whether autophagy results in cell survival or death depends on the context [29].

*Pyroptosis.* Pyroptosis involves caspase-1 mediated cell death, an atypical caspase-dependent mechanism seen in monocytes, macrophages, and dendritic cells in case of microbial infection with implications in host defence [26, 30].

*Paraptosis.* Paraptosis is cytoplasmic vacuolization initiated by swelling of mitochondria and ER. The response is mediated by mitogen-activated protein kinases (MAPKs) [31].

*Mitotic Catastrophe.* Mitotic catastrophe is a process occurring in the absence of complete mitosis. It is characterized by multinucleated enlarged cells [28] and generally marked as a cellular strategy to combat genomic instability, which is very common in cancer. The major factors involved are cell cycle-dependent kinases such as cyclin-dependent kinase 1 (cdk1), aurora kinase B, polo-like kinases (Plks); cell cycle checkpoint proteins (Chk1 and 2, p53, and Rb); Bcl-2 family proteins; and caspases [32].

*Senescence.* The outcome of senescence in cells can be visualized by tumor suppression or promotion, aging, and tissue repair, because the process is associated with inhibition of cell proliferation, aging, and cell death [33]. Cellular senescence can occur during irreversible cell cycle arrest upon encountering oncogenic stress, wherein cells become flattened, highly vacuolated, and heterochromatinized and form autophagosomes. The key players are PTEN, p53, p21, p16, and so forth [33]. In the somatic cells, telomere shortening occurs with each replicative cycle, leading to replicative senescence and ultimately cell death which may be partially due to elicitation of DNA damage response signaling. It is the normal process of aging resulting from loss of clonogenic potential. But almost 85% of human cancers show enhanced expression of telomerases [34] accounting partially for immortalization of the cancer cells. Culture stress, like substrata, serum, oxidative stress, and so forth, may also lead to senescence in* in vitro* settings [33, 35].

### 2.2. Pathways Involved in Cell Death and Their Components

For better understanding of the molecular mechanisms of the various modes of cell death mentioned above, here we have discussed the different pathways and the factors that are the main players involved in executing the cellular fates (also see Figure 1). As mentioned earlier, several signaling pathways are common in case of the cell death pathways and these involve various common players. The cellular response elicited may also overlap in certain cases. Hence, in this section, we have described the pathways one by one and intermittently discussed the involvement of the organelles in the specific contexts.

#### 2.2.1. Intrinsic Cell Death Pathways

The intrinsic death pathways are triggered by internal cellular cues and can generally be classified on the basis of their caspase dependency. Varied mitochondrial events remain associated with the ultimate outcome in either case.

*Caspase-Dependent Intrinsic Apoptotic Pathway*. The intrinsic pathway is initiated by intrinsic stimuli, like DNA damage, overload in cytosolic calcium, cellular starvation, oxidative or radiation or cytotoxic stress, and so forth, resulting in mitochondrial events determined by the Bcl-2 family proteins which have opposing roles: proapoptotic, Bax, Bak, Bad, Bcl-X_S_, Bid, Bik, Bim, and Hrk (cause mitochondrial damage) while antiapoptotic, Bcl-2, Bcl-X_L_, Bcl-W, Bfl-1 and Mcl-1 antagonize them [36, 37]. In humans, twelve caspases have been identified [38]. These are present as inactive zymogens, cleaved to produce the active caspases upon specific stimuli and function in a hierarchical fashion starting from the upstream initiator caspases (2, 8, 9, and 10) to the downstream executioner caspases (3, 6, and 7). Caspase 9 initiates mitochondrial pathways while caspase 8 and 10 trigger the death receptor mediated pathways. Under early apoptotic conditions, DNA fragmentation initiates caspase-mediated poly-ADP-ribose-polymerase (PARP) cleavage, binding the DNA fragments and blocking the access of DNA repair enzymes leading to apoptosis [39].

*Caspase-Independent Intrinsic Cell Death Pathways. *Calcium-activated calpain promotes release of apoptosis inducing factor (AIF) from mitochondria [40] and AIF translocation to the nucleus which requires PARP-1 activity [41], leading to apoptosis in a caspase-independent manner. Endonuclease G (endo G) may also participate in caspase-independent cell death pathways. Endo G is released from mitochondria under apoptotic stimuli like UV radiation or use of anti-Fas antibodies, to translocate to the nucleus, wherein it cleaves chromatin DNA into nucleosomal fragments. Endo G acts in cooperation with exonucleases and DNase I, to facilitate DNA processing. This nuclease activity may be found even in the presence of caspase inhibitors.

Granzyme A (Gzm A) also plays a role in caspase-independent apoptosis caused by massive single-stranded DNA nicking. Gzm A induces loss of mitochondrial inner membrane potential and generates reactive oxygen species (ROS). In the nucleus, ROS cleaves three members of the ER-associated DNA repair SET complex (HMG2, APE1, and SET). As a consequence, DNase NM23-H1 is activated, along with enhanced activity of other DNases due to destabilization of nuclear lamins and histone H1 [42]. Gzm C/H and K function similar to Gzm A, but their roles have not yet been fully identified [43].

*Mitochondrial Response. *The mitochondrial involvement in cell death may occur under cellular stress like nutrient deprivation or hypoxia, DNA damage, or activation of oncogenes leading to deregulated cell cycle and evasion of cell cycle checkpoints triggering aberrant cell death pathways. The Bcl-2 family proteins (Bax and Bak) oligomerize, leading to mitochondrial outer membrane permeability (MOMP). This is considered as the state of “no return.” This leads to the release of cytochrome C and/or AIF and endo G to the cytosol. Cytochrome C interacts with the apoptotic protease activating factor 1 (Apaf-1) to form the apoptosome complex [44] and subsequently activating the caspase pathway (procaspase 9, followed by caspases 3, 6, and 7). AIF and endo G trigger caspase-independent pathways. Sometimes free radicals are generated due to uncoupling of oxidative phosphorylation and diversion of electrons from the respiratory electron transport chain [45, 46]. Other mitochondrial proteins like inhibitors of apoptosis (IAPs) can inhibit the caspases via direct interaction; for example, XIAP (X-linked IAP) inhibits caspases 9, 3, and 7 [47] or IAP antagonists like SMAC/DIABLO (second mitochondrial activator of caspases/direct IAP binding protein with Low pI) or Omi/HtraA2 bind IAPs, preventing their function and favoring caspase activation.

*Endoplasmic Reticulum (ER) Stress-Induced Intrinsic Pathway.* ER stress induced by altered calcium homeostasis, glucose starvation, hypoxic stress, low redox potential, excessive or defective protein synthesis/secretion, and so forth may lead to apoptosis [48]. Upon accumulation of unfolded proteins in the ER lumen, unfolded protein response (UPR) is set off, consisting of reduction in global protein synthesis, induction of chaperones and proteins related to protein folding, and translocation of improperly folded proteins from the ER to the cytosol for proteasomal degradation [49]. Prolonged ER stress may induce autophagy (discussed later in Section 3.5); activation of “caspase 8-Bid-cytochrome C release” axis via the mitochondrial pathway (described later in the “crosstalks” subsection) [50]; or calpain-mediated activation of caspase 12 further activating caspase 9 [51].

#### 2.2.2. Extrinsic Cell Death Pathways

The extrinsic pathways are generally initiated by external stimulation of the death family of receptors and, hence, this mode is also known as the death receptor mediated extrinsic death pathway. The death receptors (DR) are a family of six members containing a conserved death domain (DD): Fas/CD95/APO-1, tumor necrosis factor receptor 1 (TNFR1), DR 3, TNF apoptosis-inducing ligand (TRAIL) R1/DR4, TRAIL R2/DR5, and DR6. Signal transduction via these receptors depends on the cellular context and stimulus, determining the outcome, which may be prosurvival, proinflammatory, apoptotic, necrotic, and so forth. The two death domain associated adaptor proteins involved here are FADD (Fas associated death domain) and TRADD (TNF receptor associated death domain). Signaling through FADD results in apoptosis while involvement of TRADD may have both apoptotic as well as nonapoptotic outcomes [52].

*Extrinsic Apoptotic Cascade. *Binding of TNF family ligands (FasL and TRAIL) to the death receptors (Fas, TNFR1, etc.) at the plasma membrane leads to recruitment of FADD, receptor interacting protein kinase 1 (RIP1) and procaspase 8 to form the death-inducing signaling complex (DISC). DISC formation triggers caspase 8 activation, ubiquitination, and degradation of RIP1 followed by caspase 3 activation and induction of apoptosis [53]. Fas may also associate with another DD associated protein Daxx (DD associated protein 6) inhibiting the FADD induced pathway and triggering JNK signaling. This leads to the induction of another discrete apoptotic cascade [54].

*Extrinsic Nonapoptotic Cascade. *When TNF-*α* binds to TNFR1, TRADD is recruited, leading to the formation of two distinct complexes, I and II [55, 56]. Complex I contains TRAF2 and 5 (TNF receptor-associated factors 2 and 5), RIP1, cIAP1 and 2; polyubiquitinating RIP1 by linking K63-Ub chains; recruiting TGF-*β* activated kinase 1 (TAK1), TAK1 binding protein 2 (TAB2), nuclear factor kappa B (NF*κ*B) essential modifier (NEMO) and I-kappa B kinase (IKK); activating NF*κ*B and leading to expression of antiapoptotic proteins, such as IAPs, and cFLIP (cellular FLICE-like inhibitory protein), and cell survival [57]. Later on, TNFR1 is internalized, leading to formation of a cytosolic complex (complex II) containing TRADD, FADD, RIP1 and 3, and procaspase 8, initiating either the extrinsic apoptotic cascade or truncating Bid (see the details later) to trigger the mitochondrial pathway. Hence, the balance between these two complexes leads to the differential outcomes [58].

*Extrinsic Necrotic Cascade or Necroptosis.* When the caspases are inactivated, a pronecrotic ripoptosome complex [59] similar to DISC is formed containing an additional member RIP3. RIP1 and 3 are activated and RIP1-RIP3 complex formation triggers production of mitochondrial ROS, PARP-1 cleavage, activation of calpains, and so forth, leading to programmed necrosis [60]. Again, DNA damage activates PARP-1, which elicits TRAF2 and RIP1 mediated JNK-1 activation to induce mitochondrial AIF release and translocation to nucleus, leading to necrotic cell death [61].

#### 2.2.3. Autophagic Cell Death and Lysosomal Response

Bcl-2 family of proteins has also been implicated in autophagic cell death, wherein, Bcl-2 can bind to Beclin-1 (a haploinsufficient tumor suppressor) and inhibit its activity. This suppresses autophagy and leads to tumorigenesis [62]. UVRAG (UV radiation resistance associated) and Bif-1 (Bax-interacting factor 1) are positive regulators of Beclin-1 promoting autophagy [63]. Bif-1 may also act via its interaction with proapoptotic Bax [64]. During starvation, Bcl-2 is phosphorylated and Beclin-1 is released inducing autophagic cell death. PARP-1 cleavage may also be associated with autophagic cell death [65]. Lysosomes are essential for autophagy. The rupture of lysosomes releases cathepsins (acid hydrolases) to the cytosol pushing cells to apoptosis or necrosis. The cathepsins trigger both caspase-dependent mitochondrial response as well as caspase-independent activation of Bax and release of AIF [66]. Some cathepsins also induce Bid mediated apoptosis [67].

#### 2.2.4. Crosstalks

In some cases, extrinsic signals may lead to low DISC formation, leading to Bid cleavage by activated caspase 8, changing the MOMP. This results in mitochondrial translocation of truncated Bid (tBid), cytochrome C release, apoptosome formation, and triggering of downstream caspase cascade, linking the intrinsic and extrinsic apoptotic pathways [50]. Formation of tBid in the cell may also result from Gzm B mediated Bid cleavage ultimately resulting in apoptosome formation as described above. Unlike the other granzymes mentioned earlier, Gzm B can also trigger a caspase-dependent apoptotic cascade and is known to cleave and activate caspases 3 and 8, while cleavage of prosurvival protein Mcl-1 leads to its inactivation [43].

DNA damaging agents may induce cell cycle arrest and induce autophagy and mitophagy, by delaying apoptosis. Both apoptosis and autophagy are regulated by the Bcl-2 family proteins [68]. Hence, apoptosis and autophagy may show synergistic effects at times, while at other times, suppression of apoptosis may lead to autophagy. Although not very clear, the two processes may be regulated by discrete DNA damage response pathways and the Bcl-2 family proteins play a crucial role in maintaining this balance. Inactivation of caspases may lead to necroptosis instead of apoptosis, while presence of necrostatin-1 (a specific inhibitor of necroptosis) may lead to reversal of necrosis to apoptosis. Sometimes programmed necrosis and autophagic cell death may occur simultaneously [60].

#### 2.2.5. Compartment-Specific Responses in Cell Death Pathways

*Nuclear Response*. The response elicited upon nuclear DNA damage is mainly via p53 activation, either triggering the transactivation of a number of Bcl-2 family proteins (Bad, Bid, Puma, and Noxa) which are effectors of mitochondrial destabilization or activation of caspase 2 and further inhibition of NF*κ*B signaling, both leading to apoptosis. Upon genotoxic stress, most commonly double-strand breaks (DSBs) activate the ATM (ataxia-telangiectasia mutated) and ATR (ataxia-telangiectasia mutated and Rad3-related) kinases, which phosphorylate and stabilize p53, blocking its ubiquitination by Mdm2 [69]. The outcome may be either G1 or G2 cell cycle arrest due to stabilization of p21 or apoptosis due to upregulation of Bax or PUMA [70]. Other kinases, like Plk-3, homeodomain interacting protein kinase 2 (HIPK-2), may also phosphorylate p53 giving apoptotic outcomes [71].

Cell cycle is a highly regulated process with multiple checkpoints arresting cells at G1/S, intra S, G2/M, mitotic spindle assembly; either for DNA repair or sending cells to death pathways if the damage is extreme; or even forcing cells to enter quiescence by exiting cell cycle (G0 phase) during starvation. Cell cycle arrest may become irreversible sending cells to senescence [72]. Rb controls the G1 checkpoint and is phosphorylated by an array of cdks to be inactivated, for transition from G1 to S phase. The mitotic checkpoint is maintained by Mad, Bub, aurora kinases, and Plks, to check the mitotic spindle formation. To circumvent aberrations in this phase, cells undergo mitotic catastrophe. Loss of control of the checkpoints leads to genomic instability, providing adaptive or selective advantage to the cancer cells [73].

*Cytosolic Response.* Akt is an important oncogenic kinase which is one of the master regulators acting upstream of many pathways involved in cell survival, proliferation, death, transcription, translation, and so forth [74]. Phosphorylation of forkhead box proteins (FOXOs) by Akt leads to their nuclear exclusion repressing proapoptotic genes—p27, Bim, and FasL. Other direct proapoptotic targets of Akt are Bad, caspase 9, Mdm2, and GSK-3*β* [75]. IKK activation induces NF*κ*B signaling to transcribe antiapoptotic proteins—Bcl-X_L_, XIAP, and so forth [76]. All the above processes inhibit apoptosis. One of the negative regulators of Akt is phosphatase and tensin homolog (PTEN), which is often deleted or mutated in cancers. While under nutrient deprivation, mTOR complex 1 is inactivated and autophagic response is initiated. Therefore, deregulation of Akt pathway is another strategy of the cancer cells to gain chemoresistance.

### 2.3. Perspectives in Cancer

Evasion of apoptosis is one of the hallmarks of cancer [77] and alterations in the apoptotic cascade may result in changes in tumor development and cancer progression [78]. Malignant cells inactivate the endogenous inducers of cell death as a strategy to block the natural cell death pathways, providing a selective survival advantage to the cancer cells [79]. There are several examples where targeting the cell death pathways in regulating the process of oncogenic progression has been used as a strategy for drug development by restoration of the endogenous autodestruction pathways [79–81]. Most of the chemotherapeutic drugs act by triggering the cell death pathways in the tumor cells [82]. A major part is by inducing apoptosis, either via the mitochondrial pathways [83] or by stimulation of the death receptor pathways [84], although involvement of the other modes of cell death has also been reported [85, 86]. Apoptosis and autophagic cell death do not elicit any immune response in the cells and hence are preferred over necrosis. While both of these processes are often found to be defective in cancer, necrosis may be found in tumors. A possible explanation may be elicitation of a persistent cytokine production helping in tumor growth leading to poor prognosis [87]. Cancer cells adapt to hypoxic stress and activate stress response pathways involving the hypoxia-inducing factors (HIFs), inducing autophagy at the hypoxic core and promoting cell survival. The response mostly depends on the dose of the chemotherapeutic stress and on the cell type [1]. Some of the strategies have been discussed below.

#### 2.3.1. Targeting Bcl-2 Family Proteins

Natural compounds, synthetic antagonists, and analogs of Bcl-2 family members have been used to regulate cell death pathways. Bid and Bax are subject to ubiquitin mediated degradation attenuating apoptosis in cancer cells (example, mitochondrial Bax is degraded in PCa cells). While this strategy is utilized by cancer cells, it has also been exploited for therapeutic purposes; for example, inhibition of the proteasomal system sensitizes chronic lymphocytic leukemia (CLL) cells to TRAIL-induced apoptosis [88].

#### 2.3.2. Targeting the Caspases

The cancer cells follow three mechanisms to negate the effect of caspases: preventing the activation of the procaspases, neutralizing active caspases, and regulating the gene expression of either caspases or their activators. There are eight members in the IAP family in humans which can directly bind to the caspases and either block their activity or mark them for ubiquitin mediated degradation. These IAPs are frequently found to be upregulated in cancers [89]. For example, c-FLIP suppresses TNF-*α* induced apoptosis via caspases 8 and 10; CARD8 (caspase recruitment domain-containing protein 8) binds to procaspase 9; XIAP inhibits caspases 3, 7, and 9. Natural antagonists of the caspases such as SMAC/DIABLO and Omi/HtrA2 compete with caspases to bind IAPs [90].

#### 2.3.3. Targeting the Tumor Suppressors

In cancers, p53 pathway is frequently inactivated by either p53 mutations or Mdm2 overexpression. In such cases, DNA damage response is elicited via ATM and ATR kinases which regulate the Chks, in turn, activating NF*κ*B, Akt, survivin, and so forth [91]. Cancer cells have evolved strategies to counteract these basic cellular mechanisms to deregulate the cell cycle and facilitate either cancer cell growth or to evade cell death. Frequent inactivating mutations or deletions in tumor suppressors like PTEN, p53, Rb, BRCA-1 and 2, p16, and ATM are associated with cancers. Premature senescence has been reported as a drug-induced tumor suppressive mechanism having a potential in cancer treatment [92]. Some tumor suppressors induce autophagy, for example, Beclin 1, UVRAG, PTEN and Bcl-2, while some oncogenic proteins like mTOR inhibit autophagy. p53 displays a dual role by both inducing and inhibiting autophagy.

## 3. DUBs Involved in Cell Death Associated Pathways Related to Cancer

**Figure 2:**
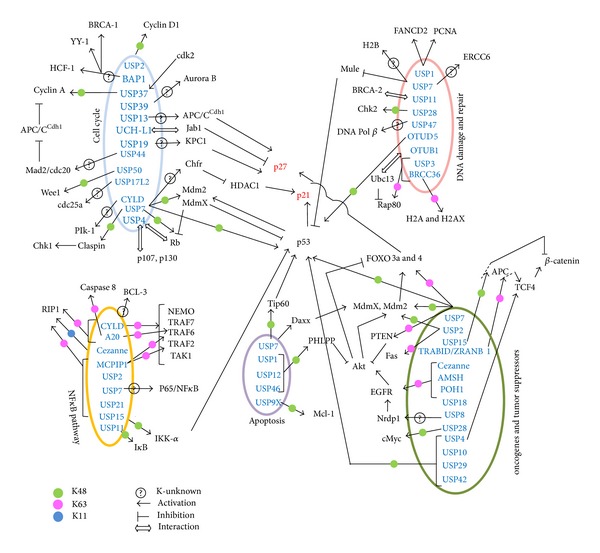
DUBs regulating cell death pathways. The figure illustrates the deubiquitinases regulating different pathways involved in cell death and cell growth in the perspective of cancer cells. The DUBs remove K48 (green), K63 (pink), and K11 (blue) linked Ub chains from their substrates. In some cases, the specific linkage remains unknown (?). The role of some of the DUBs is unclear in terms of deubiquitination of the substrate, but there is interaction (indicated by ⇔). Activation is represented by (→) and inhibition by (*⊤*).

As DUBs are integral regulatory molecules of most of the cellular functions, it has high implications in proper functioning of the cellular machineries (elaborated in other reviews [20, 21, 23, 24, 93–95]). Deubiquitinating enzymes negatively regulate the ubiquitin signaling pathway and influence both oncogenes and tumor suppressors. Due to their varied substrates, the nature of the DUBs always remains dual (both oncogenic and tumor suppressive) and their function is largely tissue-specific and context-dependent. Some of the cellular processes that are integrally related to cell death include cell cycle, DNA damage response and repair, and other signaling pathways. In this review, we emphasize on the different DUBs involved in these pathways (briefly outlined in Table 2 and Figure 2).

### 3.1. Cell Cycle

The process of cell division goes on simultaneously with cell death processes, establishing several links. Any deregulation in the continuous cycling may lead to cell cycle arrest, senescence, or death. Many DUBs such as USP7, USP13, USP39, USP44, CYLD (cylindromatosis), and BAP1 (BRCA1 associated protein-1) are associated with the different phases of cell division.

#### 3.1.1. G1, S, and G2 Phases

USP2 stabilizes cyclin D1 by direct interaction [96]. USP7 or HAUSP (herpesvirus-associated ubiquitin-specific protease) deubiquitinates SCF-*β*-TrCP mediated K48-linked Ub chains on claspin, the upstream regulator of Chk1 [97]. USP13 counteracts S phase kinase-associated protein 2 (Skp2) ubiquitination via the anaphase promoting complex/cyclosome (APC/C^Cdh1^), delaying cell cycle by accumulation of p27 [98]. USP19 deubiquitinates Kip1 ubiquitination-promoting complex protein 1 (KPC1) regulating p27^Kip1^ [99] and some KPC1 independent cell cycle regulation also exists [100]. UCH-L1 colocalizes with Jab1 sending p27^Kip1^ to proteasomal degradation, prevents senescence, and ensures proper somatic cell division [101]. USP17L2 deubiquitinates cdc25a, promoting oncogenic transformation [102].

#### 3.1.2. Spindle Assembly and Mitosis

USP39 deubiquitinates aurora B kinase maintaining spindle assembly checkpoint integrity [103] while USP44 stabilizes Mad2/Cdc20 complex inhibiting premature activation of the APC/C^Cdh1^ complex [104].

#### 3.1.3. G1/S and G2/M Checkpoints

USP7 regulates multiple cell cycle checkpoints, via deubiquitination of p53 [105] and Rb [106] or their negative regulator Mdm2 [107]. USP4, reported as an oncogenic protein, is known to interact with the pocket proteins (Rb, p107, and p130) although no deubiquitinating activity has been reported [108]. USP7 deubiquitinates checkpoint with forkhead and RING finger domains protein (Chfr), which in turn ubiquitinates histone deacetylase 1 (HDAC1), leading to upregulation of p21^Cip1/Waf1^ and induction of G1 arrest [109]. Cdk2 activates USP37 which also antagonizes APC/C^Cdh1^ complex, deubiquitinating cyclin A and entry into S phase, another G1/S checkpoint [110]. BAP1 also controls G1/S cell cycle progression by regulating BRCA-1 [111], Ying Yang 1 (YY-1), and host cell factor 1 (HCF-1) [112]. CYLD regulates Plk-1 [113] protecting G2/M checkpoint. USP50 regulates HSP90-dependent Wee1 stability preventing mitotic entry, acting as another G2/M checkpoint [114].

### 3.2. DNA Damage and Repair

DNA damage and high mutation rates are responsible for genomic instability in cancer cells. DNA damage triggers DNA repair pathways and some of the major ones in the mammalian system are mismatch repair (MMR), double strand break (DSB) repair, base excision repair (BER), nucleotide excision repair (NER), homologous recombination (HR) repair, nonhomologous end joining (NHEJ), translesion DNA synthesis (TLS), and so forth. The different pathways exist to combat the insults from a variety of DNA damage stimuli and this is known as cellular DNA damage response. These pathways are highly subject to regulation by the UPS and DUBs [115]. When the cells reach a state of chronic damage, that is, the point of no return, several other cellular responses are elicited such as apoptosis, autophagic cell death, and senescence [68].

#### 3.2.1. Double Strand Break Repair

DSB repair pathway is initiated by recruitment of BRCA-1 and p53 binding protein 1 (TP53BP1). K63-linked ubiquitin accumulates on Rap80 at the DSB foci with the concerted effect of RNF8, RNF168, and Ubc13, which are clipped off with the assistance of USP3 and BRCC36 to maintain the G2/M checkpoint. BRCC36 also hydrolyzes K63-linked Ub chains form H2A and H2AX at the sites of double strand DNA damage [116, 117]. Although OTUB1 is not catalytically involved in deubiquitinating these K63-linked chains, it may interact with Ubc13 and inhibit the E3 ligase RNF168 [118]. USP11 plays a role in the HR in response to DNA damage induced by DSBs caused by agents like bleomycin, mitomycin C, cisplatin, and so forth [119]. Although there is no evidence of BRCA-2 deubiquitination by USP11, the interaction may be involved in recruiting USP11 to the damage site [120]. USP28 stabilizes Chk2 in Chk2-p53-PUMA pathway inducing apoptosis [121]. OTUD5 also helps in stabilizing p53 and inducing apoptosis upon DNA damage signals [122].

#### 3.2.2. Base Excision Repair

BER mechanisms are elicited by genomic instability arising from DNA base lesions. DNA Pol *β* is a component of the BER complex. It is ubiquitinated by Mule and CHIP and is deubiquitinated by USP47 [123]. USP7 plays multiple roles in BER. It promotes BER by regulating chromatin remodelling by deubiquitination of H2B, though this activity was shown* in vitro*, this activity was also seen as an indirect result of Mdm2 deubiquitination by USP7 [124]. Also, DNA damage induced dephosphorylation of USP7 subjects Mule (an E3 ligase for p53) to self-ubiquitination and degradation, stabilizing p53 and activating the damage repair pathway [125]. In this context, it might be worthwhile to mention that post-translational modifications are very important in BER pathways. Apart from phosphorylation and acetylation, AP endonuclease (APE1) is also ubiquitinated, with the help of Mdm2 for degradation. This is a point of crosstalk between p53 and BER pathways [126]. As USP7 is a crucial factor in regulating the p53-Mdm2 balance in the cells, it may be speculated to play yet another role in BER response via modulation of APE1.

#### 3.2.3. Nucleotide Excision Repair

During UV radiation-mediated damage, stalling of RNA Pol IIo at DNA lesion sites is a signal for apoptosis and its removal or degradation allows the access to NER machinery. The RNA Pol II cofactors are UV-sensitivity scaffold protein A (UVSSA), ERCC6, and ERCC8. USP7 is an additional cofactor in the complex and stabilizes ERCC6 [127].

#### 3.2.4. Crosslink Repair

This mechanism involves PCNA (proliferating cell nuclear antigen) and FANCD2 (Fanconi anemia, complementation group D2) and acts at the site of fork-blocking lesions arising from interstrand crosslinks. USP1 deubiquitinates both PCNA and FANCD2 [128, 129]. Response to cell damage is generally under the control of ATM/ATR and Chk1 and 2. FANCD2 stabilization leads to activation of Chk1, the initial step in DNA damage repair [130, 131]. A number of DUBs, such as USP15, USP19, USP28, and USP34 [132, 133], act at the interface of DNA damage and cell cycle progression by DNA repair to decide the cell fate [25].

### 3.3. Apoptosis

Both apoptosis promoting and suppressing roles are displayed by the various DUBs linked to the apoptotic pathways. USP2, USP7, USP8, USP9X, USP15, USP16, USP17, USP28, CYLD, UCH-L1, A20, and so forth promote apoptosis while USP2, USP7, USP9X, USP18, and so forth suppress apoptosis [20]. USP7 deubiquitinates and stabilizes the acetyltransferase Tip60 to induce p53-dependent apoptotic pathways [134]. The opposing roles played by USP7 and Mdm2 is critical for maintaining the level of Daxx in the cancer cells [135]. In colon adenocarcinoma cells, the WDR48 and USP12 complex deubiquitinates PHLPP1 (PH domain and leucine rich repeat protein phosphatase 1) to enhance its stability, hence negatively regulating Akt activation and promoting cellular apoptosis [136]. USP1 [137] and USP46 [138] are other DUBs known to deubiquitinate PHLPP1, with a similar outcome in other cancers as well. Radiation-induced activation of USP9X deubiquitinates Mcl-1, inhibiting its degradation and apoptosis, conferring radioresistance [139].

### 3.4. Signaling Pathways Involving Key Oncogenes and Tumor Suppressors

The decision of cell death or aberrant growth leading to tumorigenesis is an outcome of the imbalance in the regulation of oncogenes and tumor suppressors. So here we have indicated the different DUBs regulating these processes.

#### 3.4.1. Signaling through Receptor Tyrosine Kinases (RTKs)

RTKs are important upstream factors in oncogenic signaling cascades and also targets for drug development. These are frequently internalized, ubiquitinated, and sorted in the endosomes leading either to lysosomal degradation or change in subcellular localization like nuclear translocation. These processes are prone to multiple aberrations in cancers with varying outcomes. AMSH (associated molecule with the SH3 domain of STAM) expression is elevated in many cancers and is capable of hydrolyzing K63-linked Ub chains from epidermal growth factor receptor (EGFR) recycling it to the plasma membrane [21, 95]. Cezanne-1 deubiquitinates and stabilizes EGFR, enhancing EGFR signaling and cancer progression [140]. USP18 also regulates EGFR [141]. POH1 regulates ErbB2, an EGFR family member [95]. USP8 regulates another EGFR family member, ErbB3 by modulating Nrdp1 (neuregulin-receptor-degradation protein-1) [142].

#### 3.4.2. Wnt/*β*-Catenin Signaling

DUBs regulate canonical Wnt signaling, by modulating *β*-catenin activity. USP4 deubiquitinases TCF4, to suppress *β*-catenin dependent transcription [143]. While USP15 deubiquitinates and stabilizes tumor suppressor adenomatous polyposis coli (APC) involved in the proteasomal degradation of *β*-catenin [144]. TRABID (TRAF-binding domain-containing protein) or ZRANB1 (zinc finger Ran-binding domain-containing protein 1) deubiquitinates APC by removing K63-linked Ub chains, inducing TCF4-mediated transcription upon Wnt stimulation [145].

#### 3.4.3. NF*κ*B Signaling

Activation of IKK leads to phosphorylation and degradation of the I*κ*Bs, promoting the nuclear translocation of NF*κ*B. K63-linked Ub-NEMO binds IKK recruiting it to complex I, activating NF*κ*B (via cIAP 1 and 2; cFLIP; and TNF-*α* and IL-8); or JNK and p38 MAPK; for antiapoptotic or proinflammatory responses, respectively, [146]. K63-linked Ub chains on RIP1 serve as a scaffold for binding of TAK1 and TAB2. Upon TNF-*α* induction, RIP1 ubiquitination may show three specific outcomes. In the first case, K48-linked ubiquitination of RIP1 may lead to targeting it for degradation and triggering the apoptotic cascade. In the second case, K63-linked ubiquitination of RIP1 may lead to association of complex I, inducing proinflammatory or antiapoptotic response. In the third case, removal of K63-linked ubiquitin chains from RIP1 may lead to the association of complex II again sending the cells to death pathway. Hence, it is clear that the ubiquitination of these factors, especially, the ubiquitin linkage of RIP1 is very important in determining cellular fate. The involvement of multiple DUBs in this pathway shows the importance of deubiquitinating enzymes in determining the cell fate via this signaling axis [95].

*CYLD. *CYLD is a negative regulator of NF*κ*B signaling contributing to oncogenesis. Knockdown of CYLD resulted in enhanced NF*κ*B signaling upon TNF-*α* stimulation [147]. The members of NF*κ*B signaling are subjected to K63-linked ubiquitination for their activation which is again deubiquitinated, as for example–CYLD deubiquitinates with NEMO, TRAF2, 6 and 7, RIP1 and TAK1 [148–150]. CYLD itself is regulated by NF*κ*B creating a negative feedback loop [151]. CYLD also inactivates BCL-3 by deubiquitination [152]. As BCL-3 is a coactivator of NF*κ*B, this inactivation of BCL-3 leads to switching in the transcriptional activity of NF*κ*B from repression to activation of cell proliferation [153, 154]. CYLD negatively regulates BCL-3 mediated NF*κ*B signaling by induction of p50-BCL3 and p52-BCL3 complexes and inhibiting cell proliferation [152].

*A20.* A20 negatively regulates NF*κ*B signaling. The targets of A20-mediated deubiquitination are K63-linked ubiquitinated TRAF6 and RIP1 [155, 156]. The enzyme A20 has been shown to have a dual role on RIP1, by acting both as a DUB (removing K63-Ub) as well as an E3 ligase (linking K48-Ub and marking it for degradation), negatively regulating the NF*κ*B signaling [156]. Apo2L induces both K48 and K63-linked polyubiquitination of caspase 8. K63-linked caspase 8 ubiquitination makes it enzymatically more potent leading to apoptosis. A20 acts as a DUB removing the K63-Ub and has been hypothesized to add K48-Ub due to its E3 ligase activity similar to its dual role as seen in case of RIP1 [157]. In B-cell lymphomas A20 is frequently inactivated and upon its restoration, cells undergo apoptosis [158].

*USP7.* USP7 is ubiquitinated by TRIM27 complex, which deubiquitinates RIP1, resulting in positive regulation of TNF-*α* induced apoptosis [58]. USP7 deubiquitinates p65-NF*κ*B by interacting with p65 at the target gene promoters, increasing their transcription [159].

*Other DUBs. *USP2a removes K63-linked Ub chains from RIP1 and TRAF2 again negatively regulating NF*κ*B signaling pathway [160]. Both USP11 and USP15 also negatively regulate and function by deubiquitinating I*κ*B to sequester NF*κ*B in the cytoplasm. USP11 stabilizes IKK-*α* to stabilize and activate p53 [161]. USP21 and Cezanne or OTUD7B negatively regulate NF*κ*B signaling by deubiquitinating and inactivating RIP1 upon TNF-*α* stimulation, providing a feedback loop in the proinflammatory signaling [162]. Cezanne has been reported to hydrolyze linear K11-Ub chains from RIP1. MCPIP1 also removes K63-Ub from RIP1 and TRAF2 and 6 but its activity is unclear [163].

#### 3.4.4. Other Oncogenic Signals

USP2 deubiquitinates both Mdm2 and MdmX. Upon androgen stimulation in prostate cancer (PCa) cells, USP2 deubiquitinates and stabilizes Mdm2 [164] and its homologue MdmX [165]. USP2a can also deubiquitinate Fas preventing apoptosis in PCa [166]. cMyc plays a pivotal role in cell growth, differentiation, and apoptosis by regulating (both activating and repressing) the transcription of proteins involved in cell cycle, survival, protein synthesis, cell adhesion, and so forth, depending on its interacting partner in forming the functional heterodimer [167]. cMyc shows multiple mutations in various cancers. The UPS mediated degradation of cMyc involves an array of E3 ligases. USP28, often overexpressed in cancers, was shown to stabilize cMyc by reversing FBW7-*α* mediated ubiquitination [168].

#### 3.4.5. Tumor Suppressors and Associated Pathways

USP7 deubiquitinates tumor suppressors p53, PTEN, FOXO, Rb, and so forth. The stabilization of p53 is triggered upon DNA damage stress, while under normal conditions Mdm2 is a better target for USP7 [107, 169]. USP7 helps in removing mono-Ub link on PTEN, facilitating its translocation back to the cytosol, which enhances tumor aggressiveness in PCa. USP7 also regulates p27^Kip1^ via deubiquitination of the FOXOs (3a and 4). FOXO 3a and 4 are deubiquitinated by USP7, to be retained in the nucleus to promote their tumor suppressive transcriptional activity [170]. In case of Rb, USP7 shows a differential deubiquitination in normal and cancer (glioma) cells depending on the Mdm2 levels [106]. Regulation of p53 is also subject to other DUBs, such as USP4, USP10, USP29, and USP42 [171]. USP2a deubiquitinates Mdm2 hence promoting p53 degradation [164].

### 3.5. DUBs as Therapeutic Targets in Cancer

Some of the conventional chemotherapeutic drugs generally rely on restoration of the endogenous cell death pathways such as activation of proapoptotic proteins like Bax, PUMA, caspases, Apaf-1, and so forth; induction of p53 apoptotic pathway by generating genotoxic stress; creating DNA damage to trigger the repair mechanisms; pharmacological inhibitors and monoclonal antibodies to target oncogenic receptors (e.g., EGFR), kinases (e.g., Akt), transcription factors (e.g., Stat3, cMyc, NF*κ*B), and so forth to regulate the major signaling pathways. In the clinical context, differentially targeting cancer cells from normal cells according to the above strategies largely depends on the dependency of cancer cells on these aberrant signals. But cellular signaling redundancy overpowers, leading to resistance. Also many drugs turn out to be carcinogenic as discussed by Apraiz et al. [172]. This can be avoided by targeting the UPS and DUBs. Some of the possibilities are restoration of tumor suppressive deubiquitinases like CYLD [173] and inhibition of oncogenic deubiquitinases like USP9X [139]. But this approach is also not that straight forward because of the dual nature of certain DUBs like USP7.

The UPS controls a large number of cell cycle proteins such as p27, Rb, cyclin D1, p53, the Bcl-2 family members, and NF*κ*B signaling [174, 175]. And as discussed earlier in Section 1, the successful use of bortezomib in multiple myeloma (MM) utilizes the aggregation of misfolded proteins in cancer cells as compared to their normal counterparts. In this cellular perspective, the principles of autophagy, that is, nutrient recycling via delivery of cytosolic contents to the lysosomes, has been found to be utilized in disposing protein aggregates, leading to the emergence of the “quality control autophagy” concept. The two ubiquitin binding proteins p62 and HDAC6 recognize the Ub-linked protein aggregates that have evaded proteasomal degradation and reroute them to the aggresome-autophagy machinery. The DUB ataxin 3 plays an important role here in remodelling the Ub-tags on protein aggregates to modify the existing signals and subsequent cell fate [176]. Combinatorial antitumoral effects of autophagic and proteasomal inhibitors have shown promises in MM [177].

The DUBs themselves are subject to different modes of regulation like transcriptional, post-translational, change in subcellular localization via multiple protein-protein interactions as discussed by Fraile et al. [20]. Some of the regulatory mechanisms are feedback loop (e.g., autoproteolysis of USP1; proteolysis of CYLD and A20), caspase 3 mediated proteolytic processing of USP7 (as reported in playing a role in thymocyte apoptosis [178]), phosphorylation (inactivates CYLD, USP8; activates A20, USP7, USP15, USP19, USP28, USP34, USP37), ubiquitination (inactivates UCH-L1, USP7, USP36), neddylation and sumoylation (inactivates USP25); ROS (modifies Cezanne); targeting of USP30 to mitochondria, or USP36 to nucleoli, and so forth. From the structural point of view, conserved catalytic sites may be blocked or conformational changes may modulate their activity (UCH-L1, OTU1, USP1, USP7, USP12, and USP46).

All these reports suggest a potential of DUBs as therapeutic targets in cancer. Till date several strategies to inhibit the DUB activity have been studied. Here we have discussed the various inhibitors of DUBs and the degree of success achieved with regard to cancer cell death. There are many small molecule inhibitors, natural compounds, and their analogs and quite a few are in preclinical trials (see details in Table 3). Some of the inhibitors are broad spectrum, while there are some which are very specific, targeting only a particular DUB. On the basis of the wide range of DUB substrates, it is very interesting to target them to induce cell death pathways in the cancer cells. But due to the highly context-dependent functioning of these DUBs, it becomes very important to understand the detailed molecular mechanism of the DUB activity in a specific pathway before targeting them with inhibitors. All the processes discussed in Section 3 can be exploited to our benefit. It may be speculated that this approach of targeting DUBs may have an edge over the conventional therapies to combat the tendency of cancer cells to acquire resistance. It is also suggestive of a combinatorial drug treatment of DUB inhibitors with the conventional cytotoxic drugs [171].

## 4. Concluding Remarks

It is well established that uncontrolled cell growth in cancer is not an outcome of abnormal cell proliferation alone but is also because of the lack of cell death, especially, the pathways that are programmed. The significance of cell death pathways in the perspective of cancer is that any deregulation in cell death leads to abnormal cell survival and proliferation, leading to oncogenesis. Hence, the knowledge of the various signaling pathways involved in cell death and their crosstalks is very important to figure out the most plausible therapeutic targets. During radio- or chemotherapy, one of the strategies exploited is the reactivation of signaling pathways, to induce cell death. The major hurdle in cancer treatment has been the redundancy in signaling pathways, favoring development of resistance against conventional therapies [179]. Hence, the requirement of focusing on alternative strategies arises.

## Figures and Tables

**Table 1:** Comparative analysis of the different modes of cell death.

Feature	Mode						
	Apoptosis	Necrosis	Autophagic cell death	Pyroptosis	Paraptosis	Mitotic catastrophe	Senescence
Programmed	Yes	Yes	Yes	No	Yes	No	No
Change in cell volume	Reduction	Increases	—	—	—	Increases	—
Chromatin condensation	Yes and nuclear fragmentation	No	No	—	—	Micronucleation	Heterochromatinization
Organelle status	Generally unaltered	Swelling of organelles	Lysosomal rupture	—	Mitochondria and ER swelling	Multinucleated cells	—
Change in membrane dynamics	Blebbing and PS flipping	Ruptured	—	—	—	—	—
Cytoplasmic material	No sequestration/release	Released due to cell rupture	Sequestered by autophagic vacuolization	—	Vacuolization seen	—	Vacuolization seen
Mitochondrial response	Occasionally	Occasionally	Occasionally	—	—	Occasionally	No
Caspase dependence	In some cases	In some cases	In some cases (caspase-1)	In some cases (caspase-1 or 7)	No	In some cases (caspase-2)	—
Immunological response	Rarely	Yes	No	—	—	—	—
Inflammatory response	Generally no	Yes	—	Yes	—	—	—
ER stress response	Yes	—	Yes	—	—	—	—
DNA damage response	Yes	Yes	Yes	—	—	Yes	Yes
Occurrence	During development, in adult tissues and pathological conditions	Physical injuries and pathological conditions	Stress response and development	In microbial infection as host defence	At times overlaps with other PCDs	Triggered by mitotic failure	Mainly stress-induced, during aging, and may involve telomere-shortening

**Table 2:** DUBs and their substrates involved in cell death pathways.

Deubiquitinase	Substrate	Hydrolyzes Ub-linkage	Relevance in cell death
Ubiquitin specific proteases			
USP1	FANCD2	—	Activates Chk1
	PCNA	—	
	PHLPP	K48	Inhibits Akt to induce apoptosis
USP2	Cyclin D1	K48	Cell cycle progression
	Mdm2	K48	Inhibition of p53
	MdmX	K48	Inhibition of p53
	Fas	K63	Inhibition of NF*κ*B signaling
USP2a	RIP1	K63	Inhibition of NF*κ*B signaling
	TRAF2	K63	Inhibition of NF*κ*B signaling
USP3 + BRCC36	Rap80	K63	Maintain G2/M checkpoints on DSBs
USP4	Rb, p107, p130	Associates	Cell cycle arrest
	TCF4	—	Suppresses *β*-catenin transcription
	p53	K48	p53 stabilization
USP7 (HAUSP)	PTEN	K63	PCa progression
	p53	K48	Apoptosis
	Rb	K48	Differential regulation
	Mdm2/MdmX	K48	Inhibits p53
	Daxx	—	Regulates Mdm2 activity under stress
	p53/Mule	—	Indirect regulation of p53
	H2B, Mdm2	—	Regulates chromatin remodelling
	Tip60	K48	p53 dependent apoptosis
	Chfr	—	Enhanced ubiquitination of HDAC1 and upregulation of p21
	ERCC6	—	Stabilizes RNA Pol II-ERCC6 complex
	Claspin	K48	Chk1 regulation
	FOXO 3a and 4	K63	Accumulation of p27
	RIP1	K63	Positive regulation of TNF-*α* induced apoptosis
	p65-NF*κ*B	—	Upregulates NF*κ*B target gene transcription
USP8	ErbB3 via Nrdp1	—	Activation of EGFR pathway
USP9X	Mcl-1	K48	Radioresistance
USP10	p53	K48	Stabilizes p53
USP11	I*κ*B	—	Inhibition of NF*κ*B signaling by sequestering NF*κ*B in the cytoplasm
USP12	PHLPP	K48	Inhibition of Akt to induce apoptosis
USP15	APC	K48	Promotes *β*-catenin
	IKK-*α*	K48	Inhibition of NF*κ*B signaling and activation of p53
USP19	KPC1	—	Accumulation of p27
USP21	RIP1	K63	Inhibition of NF*κ*B signaling
USP28	cMyc	K48	Reverses FBW7-*α* mediated ubiquitination
	Chk2	K48	Chk2-p53-PUMA apoptosis
USP29	p53	K48	Stabilization of p53
USP37	Cyclin A	K48	Induction of G1/S
USP39	Aurora B	—	SAC integrity
USP42	p53	K48	Enhances p53 stability
USP44	Mad2/cdc20	—	Inhibits APC/C^cdh1^ complex
USP46	PHLPP	K48	Inhibition of Akt to induce apoptosis
USP47	DNA Pol *β*	—	BER response
USP50	Wee1	K48	Prevents mitotic entry

Ubiquitin carboxy-terminal hydrolases			
UCH-L1	Jab1	Colocalizes	Inbihits p27
BAP1	BRCA-1	—	Induction of G1/S
	YY-1	—	Induction of G1/S
	HCF-1	—	Induction of G1/S
CYLD	Plk-1	—	G2/M protection
	NEMO	K63	Inhibition of NF*κ*B signaling
	TRAF 2, 6 and 7	K63	Inhibition of NF*κ*B signaling
	RIP1	K63	Inhibition of NF*κ*B signaling
	TAK1	K63	Inhibition of NF*κ*B signaling
	BCL-3	—	Induction of p50-BCL3 and p52-BCL3 complexes inhibiting cell proliferation

Ovarian tumor proteases			
A20	RIP1	K63	Inhibition of NF*κ*B signaling
	TRAF6	K63	Inhibition of NF*κ*B signaling
	Caspase 8	K63	Regulates caspase 8 activity
OTUB1	Ubc13	Associates	Inhibits RNF168
OTUD5	p53	K48	Induction of apoptosis
Cezanne (OTUD7B)	RIP1	K11	Inhibition of NF*κ*B signaling
	EGFR	—	Enhances EGFR signaling
TRABID/ZRANB1	APC	K63	Induction of TCF4/*β*-catenin transcription

JAMM/MPN domain-associated metallopeptidase			
AMSH	EGFR	K63	Regulates endocytic trafficking of EGFR
BRCC36	H2A, H2AX	K63	At sites of DSBs
POH1	ErbB2	—	Regulates EGFR signaling

Monocyte chemotactic protein-induced proteases			
MCPIP1	RIP1	K63	Inhibition of NF*κ*B signaling
	TRAF2 and 6	K63	Inhibition of NF*κ*B signaling

**Table 3:** Drugs targeting the DUBs.

Drug	Target	Outcome	Comments	Reference
Multiple Targets				
Betulinic acid	Multiple DUBs	Accumulates poly-Ub targets and enhances their degradation via UPS; inhibits PCa cell proliferation and induces apoptosis	Specific in action against cancer cells without affecting normal cells	[5]
Curcusone D	Multiple DUBs	ROS-induced inhibition of DUBs; growth inhibition and apoptosis of multiple myeloma (MM) cells	Shows synergistic effect with Bortezomib in MM	[6]
PR-619	Multiple DUBs	Accumulates polyubiquitinated proteins and enhances proteasomal activity	Small molecule inhibitor	[7]
Cyclopentenone prostaglandins, dibenzylideneacetone, curcumin, and shikoccin	Cellular isopept-idase	Cell death in colon cancer cells	Very broad spectrum	[8]
b-AP15	UCH-L5 USP14	Blocks proteasome function and promotes tumor cell apoptosis in preclinical models	High specificity	[9]
P22077	USP7 USP47	Induction of apoptosis involving p53 by multiple pathways (Mdm2, Claspin, Tip60, etc.)	Specific small molecule inhibitor	[7, 134]
WP1130	USP5 USP9X USP14 UCH-L1 UCH-37	Decreased Mcl-1, increased p53, stops cell proliferation and induces death in multiple myeloma (MM) and mantle cell lymphoma (MCL)	Poor solubility and pharmacokinetics	[10]
G9 (a WP1130 derivative)	USP9X USP24	Decreased Mcl-1, increased p53, induces cell death in MM, MCL and melanoma	Better solubility and lesser toxicity than WP1130	[10]

Specific Targets				
3-Amino-2-keto-7H-thieno[2,3-b]pyridin-6-one derivatives	UCH-L1	Moderately potent inhibitors	Does not show activity against other cysteine hydrolases	[11]
Pimozide and GW7647	USP1	Shows synergistic effect with cisplatin in cytotoxicity of NSCLC	Specificity is high	[12]
2-cyanopyrimidine and triazine derivatives	USP2	Specific biological data is absent	—	[8]
HBX 41,108	USP7	Activates p53 and induces apoptosis in cancer cells	Shows some cross-reactivity	[13]
HBX 19,818 and HBX 28,258	USP7	Leads to cell cycle arrest in HCT116 cells	Specific inhibition of catalytic activity	[14]
Spongiacidin C	USP7	—	A natural pyrrole alkaloid	[15]
HBX 90,397 and HBX 90,659	USP8	Induces G1 arrest and inhibits cell growth in HCT116 and PC3	Small molecule inhibitor	[16]
9-oxo-9 H-indeno[1,2-b]pyrazine-2,3-dicarbonitrile and analogs	USP8	Antiproliferative and proapoptotic in cancer cell lines	Selective inhibitor	[17]
1-[1-(4-fluorophenyl)-2,5-dimethylpyrrol-3-yl]-2-pyrrolidin-1-ylethanone)-IU1	USP14	Enhances proteasome function and accelerates proteolysis	Small molecule inhibitor	[18]

## Abbreviations

AIF: Apoptosis inducing factor
Apaf-1: Apoptotic protease activating factor 1
Cdk: Cyclin-dependent kinase
Chk: Checkpoint kinase
DISC: Death-inducing signaling complex
DUB: Deubiquitinating enzyme or deubiquitinase
Endo G: Endonuclease G
ER: Endoplasmic reticulum
FADD: Fas associated death domain
FOXO: Forkhead box protein
IAP: Inhibitor of apoptosis
JAMM: JAMM/MPN domain-associated metallopeptidase
K: Lysine
MAPK: Mitogen-activated protein kinase
MCPIP: Monocyte chemotactic protein-induced protease
MJD: Machado–Joseph disease protein domain protease
MOMP: Mitochondrial outer membrane permeability
OTU: Ovarian-tumor protease
PARP: Poly-ADP-ribose-polymerase
Pol: Polymerase
PTEN: Phosphatase and tensin homolog
RIP: Receptor interacting protein kinase
ROS: Reactive oxygen species
RTK: Receptor tyrosine kinase
TNF: Tumor necrosis factor
TNFR: TNF receptor
TRADD: TNF receptor associated death domain
TRAIL: TNF Apoptosis-Inducing Ligand
Ub: Ubiquitin
UCH: Ubiquitin carboxy-terminal hydrolase
UPS: Ubiquitin proteasome system
USP: Ubiquitin specific protease.
